# Associations of sclerostin with carotid artery atherosclerosis and all-cause mortality in Chinese patients undergoing maintenance hemodialysis

**DOI:** 10.1186/s12882-018-1046-7

**Published:** 2018-10-12

**Authors:** Aiqun Chen, Ying Sun, Ju Cui, Ban Zhao, Haitao Wang, Xianguang Chen, Yonghui Mao

**Affiliations:** 1 Department of Nephrology, Beijing Hospital, National Center of Gerontology, Beijing, People’s Republic of China; 2 The MOH Key Laboratory of Geriatrics, Beijing Hospital, National Center of Gerontology, Beijing, People’s Republic of China

**Keywords:** Carotid artery intima-media thickness, Maintenance hemodialysis, Mortality, Sclerostin

## Abstract

**Background:**

Previous clinical studies found inconsistent relationship between circulating sclerostin levels and treatment outcome in patients undergoing maintenance hemodialysis (MHD). Therefore, this study aimed to assess the associations of sclerostin with carotid artery atherosclerosis and all-cause mortality in Chinese patients undergoing MHD.

**Methods:**

This retrospective study assessed 84 patients undergoing MHD at the Nephrology Department of Beijing Hospital from January to April 2012, with a median follow-up of 61.2 months (range: 11.5 to 63 months). Carotid artery intima-media thicknesses (CIMTs) and atherosclerotic plaques were measured by B-mode Doppler ultrasound at baseline. Blood samples were collected for measuring serum sclerostin and soluble klotho (s-klotho) levels. The associations of sclerostin levels with carotid artery atherosclerosis was evaluated by correlation methods. Predictive factors of mortality were assessed by multivariate COX regression.

**Results:**

Baseline serum sclerostin averaged 162.01 pmol/L, with an interquartile range of 121.69 to 225.22 pmol/L, while CIMT values were 1.35 ± 0.39 mm. Carotid artery atherosclerotic plaques were detected in 68 subjects (81%). Subjects with sclerostin levels above the median value had higher CIMT (*p* = 0.038) and higher prevalence of atherosclerotic plaque (*p* = 0.025). During follow-up, 27 patients died; Kaplan-Meier curves indicated that subjects with high sclerostin levels (above the median value at baseline) had shorter survival (log rank *p* = 0.011). In multivariate COX regression analysis, serum sclerostin (HR, 1.095; 95% confidence interval [CI] 1.022–1.174, *p* = 0.010) and albumin (HR, 0.742; 95%CI 0.612–0.900, *p* = 0.002) levels were independent predictors of all-cause mortality.

**Conclusions:**

Sclerostin is positively associated with CIMT. In addition, patients with low baseline serum sclerostin undergoing MHD show better survival.

**Electronic supplementary material:**

The online version of this article (10.1186/s12882-018-1046-7) contains supplementary material, which is available to authorized users.

## Background

Cardiovascular disease is the leading cause of mortality in patients undergoing maintenance hemodialysis (MHD), with a prevalence 3–10 times higher in such patients compared with the general population [1, 2]. Higher cardiovascular risk in patients undergoing MHD is partly explained by chronic kidney disease-mineral bone disorder (CKD-MBD), in which sclerostin plays an important role [3].

The Wnt-β-catenin pathway plays a crucial role in skeletal development and bone mass. Dysregulated Wnt-β-catenin pathway is also involved in cancer, aortic valve calcification, several bone-related phenotypes, and CKD-MBD [4, 5]. Indeed, the Wnt-signaling pathway is involved in stimulating osteoblast proliferation, differentiation, and bone formation [6]. Sclerostin, the product of the SOST gene [7], is an antagonist of the Wnt pathway [4, 5] and is involved in the crosstalk among kidneys, bones, and vasculature [5]. Sclerostin is a soluble inhibitor of Wnt-β-catenin (canonical) signaling pathway, that reduces osteoblastogenesis and promotes osteoblast and osteocyte apoptosis thus exerting a powerful anti-anabolic effect [5]. Interestingly, Pelletier et al. [8] found that serum sclerostin levels are higher in CKD patients compared with the general population, and start to increase in CKD stage III. Sclerostin gradually increases as renal function declines, and is inversely correlated with glomerular filtration rate (GFR; calculated by insulin clearance) [8]. In addition to renal retention [9], higher sclerostin in CKD may also be related to enhanced production by bone cells [3, 10]. Meanwhile, the Wnt-signaling pathway regulates vascular tissue development and endothelial cell differentiation, and is closely associated with atherosclerosis [11, 12]. Importantly, sclerostin was detected in non-uremic and uremic aortic valve calcification (AVC) and skin calciphylaxis tissues by immunohistochemistry (IHC) [13–15]. The above findings suggest a role for sclerostin in cardiovascular disease.

Recently, Viaene et al. [16] and Drechsler et al. [17] demonstrated that high serum sclerostin levels are associated with improved survival in patients undergoing MHD. But other studies indicated that high serum sclerostin is associated with higher mortality in non-dialysis CKD and MHD patients [18–20]. Based on these discrepant findings, the present study aimed to assess whether carotid artery atherosclerosis is associated with sclerostin in patients undergoing MHD. Since treatment outcome may depend on patient characteristics and follow up duration, we also determined the association of sclerostin and all-cause mortality in Chinese patients undergoing MHD.

## Methods

### Study population

The present study was a secondary analysis of a cross-sectional study conducted in 2012 that assessed 88 patients undergoing MHD [21], whose baseline serum samples were stored at − 80 °C; 4 samples were lost. Therefore, this study retrospectively assessed 84 patients undergoing MHD at the Nephrology Department of Beijing Hospital in January–April 2012. Inclusion criteria were: age ≥ 18 years, dialysis vintage ≥3 months, KT/V (urea nitrogen removal index) > 1.2, and availability of serum samples for sclerostin measurement. Patients with malignancies, autoimmune diseases, active liver disease, fractures, and/or acute and chronic infections were excluded. All subjects underwent hemodialysis for 4 h with standard bicarbonate dialysate (3 times per week), with a few subjects submitted to hemofiltration 1–2 times monthly. The dialyzer membrane was polymethylmethacrylate (P-MMA) or polysulfone (PS), with an area of 1.4–1.6 m^2^. Heparin anticoagulation was employed, with vascular access via an arteriovenous fistula. The blood flow rate was 200–300 mL/min, for a dialysate flow of 500 mL/min. The study was performed according to the Declaration of Helsinki and approved by the ethics committee of Beijing Hospital (ethical approval number: 2016BJYYEC-110-01). Written informed consent was obtained from all patients.

### Clinical and biochemical parameters

Age, sex, history of diabetes, and ESRD causes were collected by reviewing medical records. Blood samples were collected at baseline, before hemodialysis initiation. We used the first hemodialysis session of a given patient after January 2012 and each patient was assessed once. Serum urea, creatinine, uric acid, total serum calcium, phosphorus, albumin, triglycerides, total cholesterol, low-density lipoprotein cholesterol (LDL-C), high-density lipoprotein cholesterol (HDL-C), alkaline phosphatase (AP), high-sensitivity C-reactive protein (hs-CRP), and hemoglobin amounts were measured by standard laboratory methods. All routine assays were performed at the central laboratory of the hospital. Then, serum samples were stored at − 80 °C until further analysis. Soluble klotho (s-klotho) levels were assessed in August 2012 with a specific enzyme-linked immunosorbent assay (ELISA) kit (R&D Systems, Inc., Minneapolis, MN, USA), according to the manufacturer’s instructions. The range of the assay standard curve is 8 to 150 U/L. Sclerostin amounts were measured in October 2016 with an ELISA kit from Biomedica (Austria) based on the manufacturer’s protocol. The intra-assay and inter-assay coefficients of variation are, according to the manufacturer, ≤7% and ≤ 10%, respectively. The median sclerostin value in apparently healthy individuals is 24.14 pmol/L. The range of the assay standard curve is 0 to 240 pmol/L. All ELISAs and non-routine assays were performed by the same operator.

### B-mode and Doppler ultrasound of the common carotid arteries

B-mode and Doppler ultrasound of the common carotid arteries was performed at baseline. With the patient in the supine position with the head tilted backward, the common carotid arteries were detected bilaterally using color Doppler ultrasound at a frequency of 5–10 MHz (Philips model IU-22). Bilateral images of the common carotid arteries were obtained by an experienced sonographer, with each measurement repeated twice. The CIMT was obtained just before the carotid artery bulb, and defined as the vertical distance between the lumen-intima and media-adventitia interfaces on the posterior wall of the carotid artery. The results were expressed as maximum values obtained for the left and right arteries. Atherosclerotic plaque was defined as CIMT exceeding 1.5 mm [22].

### Follow-up and outcome analysis

All patients were followed-up until April 30, 2017. The primary endpoint was all-cause mortality. The time and cause of death were also recorded for each deceased patient.

### Statistical analyses

Statistical analyses were performed with the SPSS 20.0 software. Normally distributed variables were expressed as mean ± standard deviation (SD), and non-normally distributed variables as median with 25th and 75th percentiles. Differences between groups were compared by Student’s t test or Mann-Whitney U test. Categorical data were presented as percentage, and assessed by the Chi-square test. The Spearman method was used to estimate the correlation between sclerostin and other parameters. The Kaplan-Meier method was used to assess all-cause mortality. The associations of baseline indices with patient survival were assessed by Cox multivariate regression analysis. *P* < 0.05 was considered statistically significant.

## Results

### Patient baseline characteristics

The clinical and demographic characteristics of the cohort are shown in Table 1. The original dataset of the study is provided in Additional file 1. A total of 84 subjects (50 males and 34 females; mean age, 63.9 ± 11.5 years; age range, 39–87 years) were enrolled, including 28 who had diabetes. ESRD causes were chronic glomerulonephritis (*n* = 34, 40.5%), diabetic nephropathy (*n* = 20, 23.8%), hypertensive renal disease (*n* = 10, 11.9%), chronic interstitial nephritis (*n* = 10, 11.9%), polycystic kidney disease (*n* = 7, 8.3%), and other diseases (*n* = 3, 3.6%).

**Table 1:** Demographic characteristics of all patients, and comparisons between the high and low sclerostin groups

Variable	All patients *n* = 84	Scl < 162.01 pmol/L (*n* = 42)	Scl > 162.01 pmol/L (*n* = 42)	*P* value
Male/female	50/34	22/20	28/14	0.133
Age (years)	63.9 ± 11.5	61.5 ± 12.6	66.2 ± 10.0	0.062
Diabetes, n (%)	28 (33.33)	13 (30.95)	15 (35.71)	0.409
Cardiovascular history, n (%)	44 (52.4)	22 (52.4%)	22 (52.4%)	1.000
BMI (kg/m^2^)	23.59 ± 3.32	23.57 ± 3.85	23.61 ± 2.73	0.953
Systolic BP (mmHg)	149 ± 19	146 ± 17	150 ± 20	0.374
Diastolic BP (mmHg)	79 ± 11	79 ± 13	79 ± 9	0.898
Dialysis vintage, months	57.6 (27.3–85.5)	51.5 (21.2–84.4)	62.3 (38.7–92.1)	0.207
CIMT (mm)	1.35 ± 0.39	1.26 ± 0.38	1.44 ± 0.39	0.038
Plaque, n (%)	68 (80.95)	30 (71.43)	38 (90.48)	0.025
Sclerostin (pmol/L)	162.01 (121.69–225.22)	121.75 (91.03–145.44)	225.01 (194.08–250.27)	< 0.001
s-klotho (U/L)	41.27 (28.12–72.27)	44.88 (29.90–77.19)	39.37 (26.57–51.70)	0.156
PTH (10–69 pg/mL)^a^	485.50 (255.75–730.50)	498.00 (260.25–843.50)	450.50 (245.75–705.75)	0.691
Hemoglobin (120–160 g/L)^a^	114.00 (101.75–123.75)	116.00 (100.00–125.25)	113.50 (106.00–121.25)	0.585
Albumin (34–48 g/L)^a^	41.00 (39.25–42.00)	41 (39–42)	41 (40–42)	0.521
Creatinine (59–104 μmol/L)^a^	922.08 ± 258.78	917.55 ± 298.97	926.62 ± 214.81	0.847
Uric acid (208–428 μmol/L)^a^	392.27 ± 84.85	407.81 ± 91.01	376.74 ± 76.13	0.093
Phosphate (0.92–1.62 mmol/L)^a^	1.80 ± 0.53	1.77 ± 0.52	1.84 ± 0.54	0.547
Calcium (2.03–2.54 mmol/L)^a^	2.20 ± 0.22	2.18 ± 0.22	2.21 ± 0.23	0.567
Cholesterol (< 5.2 mmol/L)^a^	4.21 ± 0.92	4.29 ± 1.00	4.13 ± 0.83	0.440
LDL-C (< 3.2 mmol/L)^a^	2.31 ± 0.66	2.41 ± 0.70	2.20 ± 0.62	0.147
HDL-C (> 1.04 mmol/L)^a^	1.02 ± 0.27	1.00 ± 0.25	1.05 ± 0.30	0.379
Alkaline phosphatase (30–130 U/L)^a^	103.00 (75.50–135.50)	109.50 (76.50–159.50)	99.00 (74.75–118.75)	0.263
Kt/V	1.32 ± 0.31	1.34 ± 0.32	1.29 ± 0.29	0.414
hs-CRP (0-3 mg/L)^a^	3.18 (1.65–6.68)	3.42 (1.86–6.25)	2.31 (1.49–7.22)	0.351
Anti-hypertensive drug (n, %)	71 (84.5)	35 (83.3)	36 (85.7)	0.763
Statin (n, %)	20 (23.8)	8 (19.1)	12 (28.6)	0.306
Calcium-based phosphate binders (n, %)	63 (75.0)	30 (71.4)	33 (78.6)	0.450
Calcitriol (n, %)	49 (58.3)	27 (64.3)	22 (52.4)	0.268

^a^Normal reference values are shown between brackets. Note: no reference value for s-Kloto is provided by the kit’s manufacturer

Normally distributed variables are mean ± standard deviation; non-normally distributed variables are median with 25 and 75% interquartile ranges in parentheses. *CIMT* carotid artery intima-media thickness, *iPTH* intact parathyroid hormone, *sKlotho* soluble klotho, *LDL-C* low-density lipoprotein cholesterol, *HDL-C* high-density lipoprotein cholesterol, *Hs-CRP* high-sensitivity C-reactive protein

Men had higher sclerostin levels compared with women (191.39 vs. 155.82 pmol/L; *p* = 0.031). Although males showed higher CIMT values in comparison with females, the difference was not statistically significant (1.41 ± 0.40 vs. 1.26 ± 0.35 mm, *p* = 0.062). Diabetic subjects had higher CIMT values compared with patients without diabetes (1.50 ± 0.39 vs. 1.28 ± 0.37 mm, *p* = 0.012). Sclerostin levels between the patients with and without diabetes were comparable (174.24 vs. 160.33 pmol/L; median, *p* = 0.718).

### Differences between the high and low sclerostin groups

Since the sclerostin levels were not normally distributed, non-parametric analyses were used and the subjects (*n* = 84) were divided into two groups according to the median sclerostin level (162.01 pmol/L) into the “high” and “low” sclerostin groups, as in previous studies [16, 19]. The high sclerostin group showed higher CIMT (1.44 ± 0.39 vs. 1.26 ± 0.38 mm, *p* = 0.038) and higher prevalence of atherosclerotic plaques (90.48 vs. 71.43%, *p* = 0.025), compared with the low sclerostin group (Table 1).

### Association of plasma sclerostin with demographic characteristics and clinical parameters

There was a significant positive correlation between sclerostin and CIMT (Spearman rank *r* = 0.262; *p* = 0.016), and a negative association of sclerostin with s-klotho (Spearman rank *r* = − 0.217; *p* = 0.047). After adjustment for age, gender, and diabetes, sclerostin was still independently associated with CIMT (OR = 1.161, 95%CI: 1.046–1.288, *p* = 0.005). A positive correlation was obtained between sclerostin and age (Spearman rank *r* = 0.195; *p* = 0.075) although statistical significance was not reached. There were no significant associations of sclerostin with iPTH and AP.

### Differences between survivors and non-survivors

During the follow-up period, 27 subjects died and 18 out of 27 deaths were cardiovascular disease-related; none was lost to follow-up or received kidney transplant. Non-survivors were older (68.6 ± 11.1 vs. 61.6 ± 11.1 years, *p* = 0.009), had a higher prevalence of diabetes (51.85 vs. 24.56%, *p* = 0.014), and showed higher CIMT (1.51 ± 0.40 vs. 1.27 ± 0.37 mm, *p* = 0.011), higher sclerostin levels (217.30 vs. 152.34 pmol/L, *p* = 0.042), and lower uric acid amounts (361.93 ± 60.30 μmol/L vs. 406.65 ± 91.25 μmol/L, *p* = 0.023), compared with survivors (Table 2). Meanwhile, non-survivors showed trends of higher prevalence of carotid artery atherosclerotic plaques (92.59 vs. 75.44%, *p* = 0.053) and lower albumin levels (40 vs. 41 g/L, median, *p* = 0.060) compared with the survivor group (Table 2).

**Table 2:** Comparisons between the survivor and non-survivor groups

	Non-survival group *n* = 27	Survival group *n* = 57	*P* value
Male/female	19/8	31/26	0.123
Age (years)	68.6 ± 11.1	61.6 ± 11.1	0.009
Diabetes, n (%)	14 (51.85)	14 (24.56)	0.014
Cardiovascular history, n (%)	21 (77.78%)	19 (33.33%)	0.002
BMI (kg/m^2^)	22.92 ± 3.35	23.9 ± 3.29	0.213
Systolic BP (mmHg)	152 ± 25	146 ± 14	0.278
Diastolic BP (mmHg)	77 ± 15	80 ± 9	0.337
Dialysis vintage (months)	51.0 (35.8–80.6)	63.6 (24.8–88.45)	0.723
CIMT (mm)	1.51 ± 0.40	1.27 ± 0.37	0.011
Plaque, n (%)	25 (92.59)	43 (75.44)	0.053
Sclerostin (pmol/L)	217.30 (141.37–239.73)	152.34 (111.80–201.15)	0.042
PTH (pg/mL)	482.00 (264.00–650.00)	489.00 (243.50–911.50)	0.438
s-klotho (U/L)	42.45 (28.03–61.52)	41.21 (28.21–74.91)	0.730
Hemoglobin (g/L)	112.00 (96.00–122.00)	115.00 (108.50–125.00)	0.237
Albumin (g/L)	40 (38–42)	41 (40–42)	0.060
Creatinine (μmol/L)	864.85 ± 220.10	949.19 ± 272.83	0.164
Uric acid (μmol/L)	361.93 ± 60.30	406.65 ± 91.25	0.023
Phosphate (mmol/L)	1.80 ± 0.53	1.80 ± 0.53	0.966
Calcium (mmol/L)	2.13 ± 0.22	2.23 ± 0.22	0.064
Cholesterol (mmol/L)	4.00 ± 0.91	4.31 ± 0.91	0.156
LDL-C (mmol/L)	2.15 ± 0.60	2.38 ± 0.68	0.139
HDL-C (mmol/L)	1.02 ± 0.25	1.03 ± 0.29	0.929
Alkaline phosphatase (U/L)	104.00 (79.00–161.00)	99.00 (73.50–126.50)	0.294
KT/UV	1.30 ± 0.32	1.33 ± 0.30	0.722
hs-CRP (mg/L)	3.18 (1.86–9.27)	2.85 (1.51–6.47)	0.449

Normally distributed variables are mean ± standard deviation; non-normally distributed variables are median with 25 and 75% interquartile ranges in parentheses. *CIMT* carotid artery intima-media thickness, *iPTH* intact parathyroid hormone, *sKlotho* soluble klotho, *LDL-C* low-density lipoprotein cholesterol, *HDL-C* high-density lipoprotein cholesterol, *Hs-CRP* high-sensitivity C-reactive protein

### Association of sclerostin with all-cause mortality

**Fig. 1:**
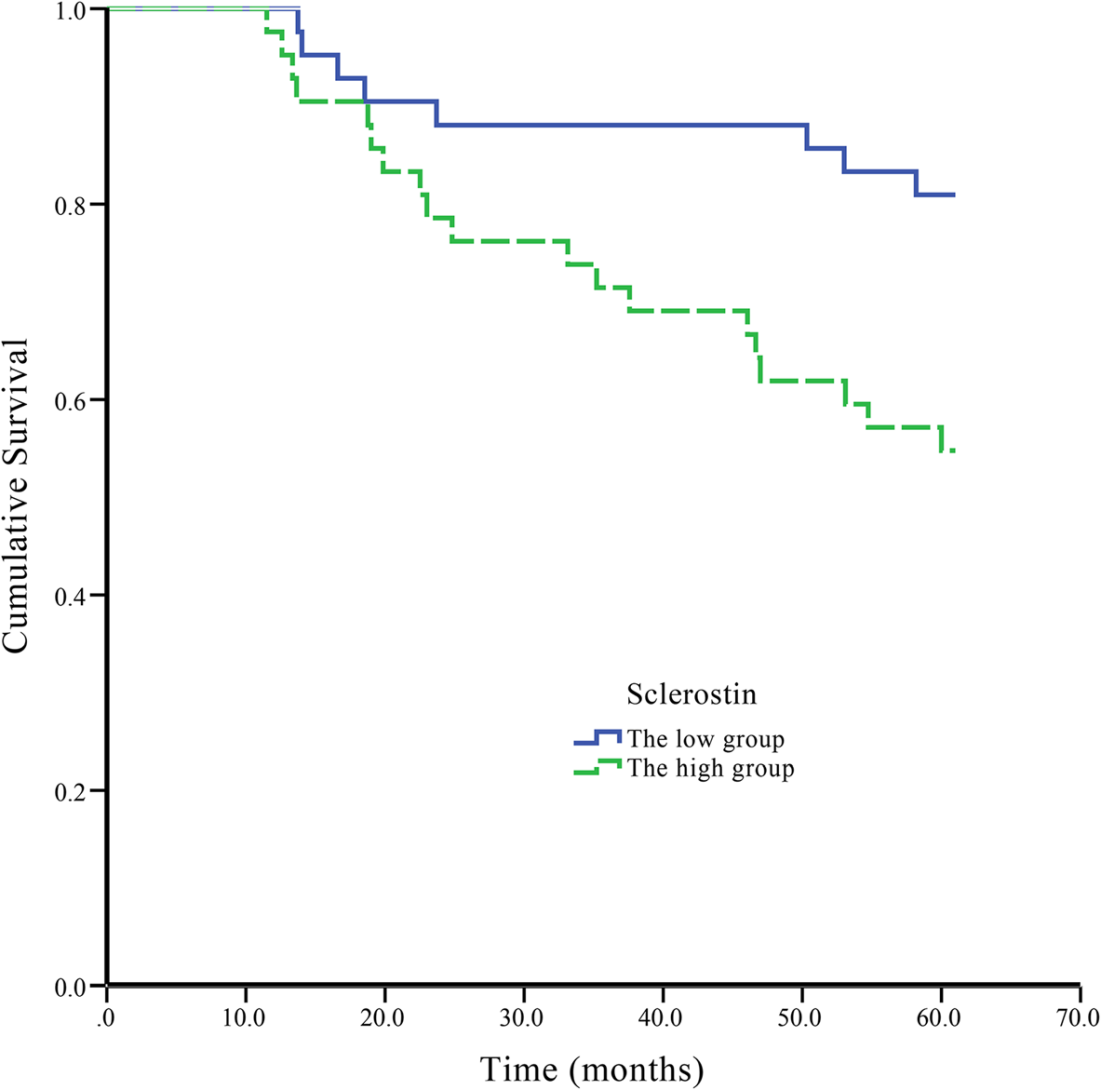
Kaplan-Meier survival curves of all-cause mortality for the low and high sclerostin groups. (Log rank *p* = 0.011)

The 84 patients were divided into two groups according to the median sclerostin level (162.01 pmol/L). Kaplan-Meier curves (Fig. 1) showed that the high sclerostin group had reduced survival (log rank *p* = 0.011) compared with the low sclerostin group. The follow-up ranged from 11.5 to 63 months (median, 61.2 months; IQR, 47.8–62.3 months). In the low and high sclerostin groups, 1-year survival was 100% and 97.6%, respectively; 3-year survival was 88.1% and 71.4%, respectively; and 5-year survival was 80.1% and 54.8%, respectively. The associations of all-cause death with various parameters were assessed by multivariate Cox regression analysis. Based on univariate analysis (Table 3), age, albumin, uric acid, sclerostin, CIMT, diabetes and history of cardiovascular disease (*p* < 0.05) were included. The results indicated that serum sclerostin (HR, 1.095; 95% confidence interval [CI] 1.022–1.174, *p* = 0.010) and albumin (HR, 0.742; 95%CI 0.612–0.900, *p* = 0.002) levels were independent predictors of all-cause mortality.

**Table 3:** Univariate and multivariate analysis for all-cause death in patients undergoing maintenance hemodialysis

Variable	HR	95.0% CI	*P* value
Univariate analysis			
Age (per year)	1.046	1.010–1.085	0.013
Diabetes (Y versus N)	2.760	1.293–5.892	0.009
Cardiovascular history (Y versus N)	3.895	1.570–9.666	0.003
Hemoglobin (1 g/L)	0.985	0.960–1.009	0.221
Creatinine (1 μmol/L)	0.999	0998–1.000	0.143
Uric acid (1 μmol/L)	0.995	0.990–1.000	0.033
Albumin (1 g/L)	0.775	0.649–0.925	0.005
Phosphate (1 mmol/L)	0.993	0.477–2.066	0.984
Hs-CRP (1 mg/L)	1.044	0.968–1.126	0.263
Sclerostin (10 pmol/L)	1.068	1.000–1.140	0.049
IMT (0.1 mm)	1.154	1.049–1.269	0.003
PTH (1 pg/mL)	1.000	0.999–1.000	0.431
Alkaline phosphatase (1 U/L)	1.000	0.998–1.002	0.934
Plaque (Y versus N)	3.559	0.843–15.033	0.084
Multivariate analysis			
All-cause mortality			
Sclerostin (10 pmol/L)	1.095	1.022–1.174	0.010
Albumin (1 g/L)	0.742	0.612–0.900	0.002

### Association of sclerostin with cardiovascular death

Next, the associations of various indices with cardiovascular death were evaluated. Age (*p* = 0.004), diabetes (*p* = 0.015), and CIMT (*p* = 0.001) showed significant associations in univariate analyses (Table 4), and were entered in the multivariate analysis. Interestingly, CIMT (HR = 1.158; 95%CI 1.017–1.320; *p* = 0.027) and age (HR = 1.063; 95%CI 1.011–1.118; *p* = 0.016) were independent predictive factors of cardiovascular death in these patients.

**Table 4:** Univariate and multivariate analysis of cardiovascular death in patients undergoing maintenance hemodialysis

Variable	HR	95.0% CI	p
Univariate analysis			
Age (per year)	1.072	1.022–1.124	0.004
Diabetes (Y versus N)	3.185	1.252–8.105	0.015
Sex (Male versus Female)	2.547	0.838–7.740	0.099
Hemoglobin (1 g/L)	1.005	0.971–1.039	0.794
Creatinine (1 μmol/L)	0.999	0997–1.001	0.395
Uric acid (1 μmol/L)	0.995	0.985–1.000	0.072
Albumin (1 g/L)	0.837	0.672–1.042	0.111
Phosphate (1 mmol/L)	0.914	0.370–2.257	0.845
Hs-CRP (1 mg/L)	1.006	0.907–1.116	0.907
Sclerostin (10 pmol/L)	1.060	0.993–1.167	0.074
I MT (0.1 mm)	1.208	1.077–1.355	0.001
PTH (1 pg/mL)	0.999	0.998–1.000	0.159
Alkaline phosphatase (1 U/L)	1.000	0.998–1.002	0.903
Plaque (Y versus N)	29.64	0.248–3536.44	0.165
Multivariate analysis			
Cardiovascular mortality			
IMT (0.1 mm)	1.158	1.017–1.320	0.027
Age (per year)	1.063	1.011–1.118	0.016

## Discussion

The association of serum sclerostin levels with survival in patients undergoing MHD remain unclear. This study revealed a positive correlation between sclerostin and CIMT, and patients with low serum sclerostin levels had improved survival. In addition, serum sclerostin and albumin levels were independent predictors of all-cause mortality in patients undergoing MHD.

Previous studies reported that serum sclerostin gradually increases from non-uremic controls without overt calcification-related cardiovascular disease to those with AVC, and from uremic patients without AVC to their counterparts with AVC [13, 15]. In uremia, vascular smooth muscle cells (VSMCs) are affected by calcium, phosphorus, stress, and other factors, and undergo osteogenic and/or chondrogenic differentiation, to form mature osteocyte-like cells [23]. Sclerostin is mainly derived from osteocytes, and it is speculated that elevated serum sclerostin might be partly derived from calcified vasculature [13]. Recently, human atherosclerotic sites were shown to display sclerostin deposition detectable by immunohistochemistry [3]. Recent clinical studies revealed that serum sclerostin levels are higher in patients with carotid artery atherosclerotic plaques undergoing MHD, with sclerostin independently associated with CIMT [24]. These findings corroborated the present results. Nevertheless, the exact biological significance of high sclerostin levels are currently unknown and the threshold for considering sclerostin levels high in patients with CKD is currently not defined [5, 25]. Nevertheless, it is known that sclerostin is a major player in the kidney-bone-vasculature axis and that it is involved in calcifications. Further in vivo studies are necessary to determine its exact role in atherosclerosis and the involvement of the Wnt-β-cathenin axis in the process [5].

The term “klotho” refers to a-klotho, which has two main types: membrane and soluble (the most abundant in humans) [26]. In cultured rat vascular smooth muscle cells (VSMCs), s-klotho controls cell differentiation and dedifferentiation by regulating the smooth muscle cell marker protein 22-a (SM22-a) and the mineralization marker Runx2, with a direct inhibitory effect on soft tissue calcification [26]. Clinical studies have also shown that serum klotho levels are inversely associated with the severity of coronary artery disease in patients with normal kidney function and abdominal aortic calcification (AAC) undergoing MHD [27, 28]. In this study, we found that sclerostin was inversely associated with s-klotho. Taken together, these findings suggest that vascular calcification is negatively associated with serum s-klotho and positively correlated with serum sclerostin concentrations.

As shown above, patients with low baseline sclerostin levels had higher survival rate compared with those of the high sclerostin group, consistent with previous findings in non-dialysis CKD and MHD patients [18–20]. The association of sclerostin with mortality may reflect its role in vascular calcification. Studies in the CKD-2 mouse model support this notion; indeed, CKD-2 mice have higher levels of DKK1 (another Wnt-signaling inhibitor) and sclerostin amounts compared with non-CKD controls, and DKK1 inhibition by a monoclonal antibody ameliorates vascular calcification and decreases sclerostin levels [29]. Other investigators suggested that high serum sclerostin levels are associated with improved survival in patients undergoing MHD [16, 17]. High sclerostin in calcified vasculature may be a defensive response in order to limit further mineralization [30]. Some authors suggested the possibility that sclerostin has an anti-calcific effect by inhibiting ALP activity; ALP can inactivate inorganic pyrophosphate, and inorganic pyrophosphate is a potent inhibitor of calcification [31, 32]. Although sclerostin was shown above to be an independent predictor of all cause mortality, it was not associated with CV-related death, indicating that other mechanisms may be involved as well. The discrepant findings among studies assessing the role of sclerostin in patients undergoing MHD may be attributable to demographic characteristics (patients of different ethnicities), sample size (the lack of association may be due to insufficient sample size), or different sclerostin detection methods (sensitivity). Therefore, whether sclerostin is pro- or anti-calcification in the context of MHD remains unclear.

The current study showed that male patients had higher sclerostin levels than their female counterparts, corroborating previous studies [24, 33]. Possible explanations include higher bone mass (sclerostin is almost exclusively secreted by osteocytes) and lower estrogen levels (estrogens reduce serum sclerostin levels) in men [33]. Serum sclerostin is positively associated with age [16, 17, 33]. The present study found such a trend, but statistical significance was not reached; this may be attributed to the limited sample size.

Limitations of this study should be mentioned. First, the sample size was relatively small. In addition, it was a single-center study of a Chinese population. Therefore, the generalizability of the current findings to other ethnicities requires further validation. Finally, histomorphometric data of the bone were lacking.

## Conclusions

In summary, sclerostin is related to atherosclerosis and all-cause mortality in patients undergoing MHD. Whether sclerostin is only a predictor or a significant risk factor for all-cause mortality remains unclear, and certainly worthy of further research.

## Additional file

Additional file 1: **Datasheet S1**. Original dataset. (XLS 51 kb)

## Abbreviations

AAC: Abdominal aortic calcification
AP: Alkaline phosphatase
AVC: Aortic valve calcification
CI: Confidence interval
CIMTs: Carotid artery intima-media thicknesses
CKD-MBD: Chronic kidney disease–mineral bone disorder
ELISA: Enzyme-linked immunosorbent assay
GFR: Glomerular filtration rate
HDL-C: High-density lipoprotein cholesterol
Hs-CRP: High-sensitivity C-reactive protein
IHC: Immunohistochemistry
LDL-C: Low-density lipoprotein cholesterol
MHD: Maintenance hemodialysis
P-MMA: Polymethylmethacrylate
PS: Polysulfone
SD: Standard deviation
s-klotho: soluble klotho
SM22-a: Smooth muscle cell marker protein 22-a
VSMCs: Vascular smooth muscle cells
